# Supplementary material for: Fröhlich interaction dominated by a single phonon mode in CsPbBr3
Source: Nat Commun. 2021 Oct 6;12:5844. doi: 10.1038/s41467-021-26192-0 (PMC8494801; doi:10.1038/s41467-021-26192-0)
Supplement: Supplementary file 1 — Supplementary Information [file 41467_2021_26192_MOESM1_ESM.pdf]

Fröhlich Interaction Dominated by a Single Phonon Mode in $CsPbBr_3$ – Supplementary Information

Claudiu M. Iaru[1,*], Annalisa Brodu[2], Niels J. J. van Hoof[1], Stan E. T. ter Huurne[1], Jonathan Buhot[3,4], Federico Montanarella[2], Sophia Buhbut[2], Peter C. M. Christianen[4], Daniël Vanmaekelbergh[2], Celso de Mello Donega[2], Jaime Gòmez Rivas[1], Paul M. Koenraad[1], and Andrei Yu. Silov[1,*]

[1]Department of Applied Physics and Institute for Photonic Integration,, Eindhoven University of Technology, P.O. Box 513, 5600 MB, Eindhoven, The Netherlands
[2]Condensed Matter and Interfaces, Debye Institute for Nanomaterials Science, , Utrecht University, Princetonplein 1, 3508 TA, Utrecht, the Netherlands
[3]HH Wills Laboratory, University of Bristol, Bristol, BS8 1TL, UK
[4]High Field Magnet Laboratory (HFML - EMFL), Radboud University,, 6525 ED Nijmegen, The Netherlands
[*]Email: C.M.Iaru@tue.nl, A.Y.Silov@tue.nl

September 16, 2021

Supplementary Note 1: Characterization of $CsPbBr_3$ NCs

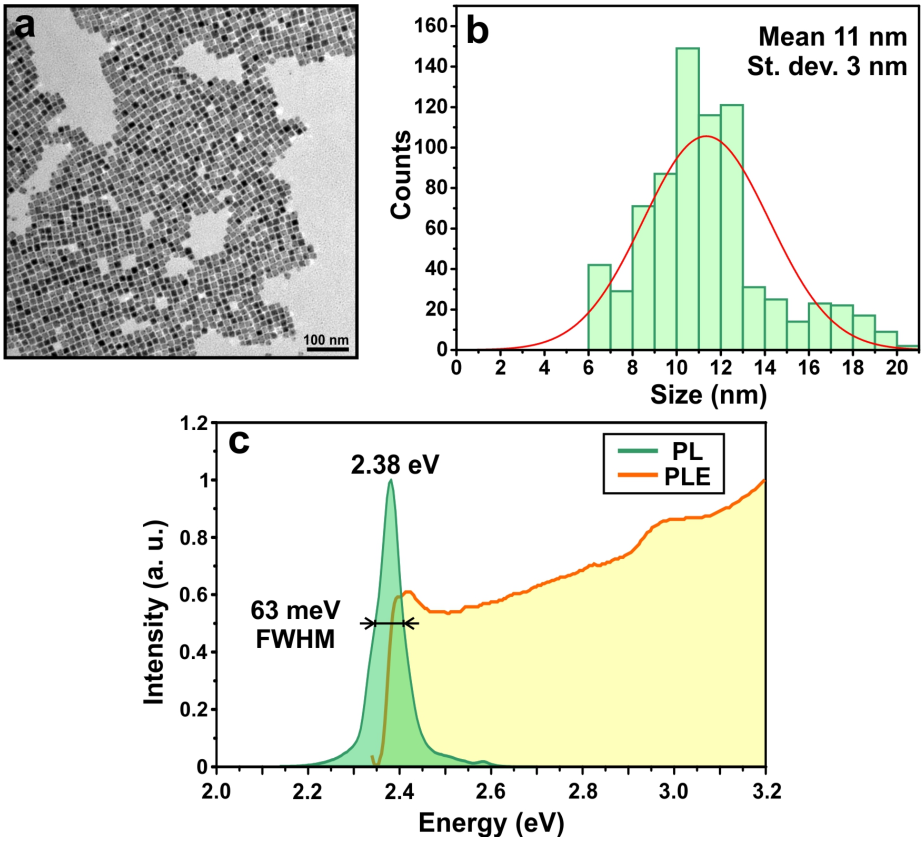

Supplementary Figure 1: Analysis of $CsPbBr_3$ NCs used in MPRS experiment. (a),(b) Representative TEM image and corresponding particle size analysis, showing an average NC size of 11 ± 3 nm. (c) 4 K PL/PLE measurements showing an emission energy of 2.38 eV with a FWHM of 63 meV. Note that the NC dimensions are several times larger than the estimated CsPbBr Bohr radius,[1] and the bulk emission energy for $CsPbBr_3$ is around 2.29 eV,[2] meaning that quantum confinement is remains relatively weak in the current sample.

Supplementary Note 2: Fröhlich Interaction: Weak, Strong, and Intermediate Coupling

Weak coupling corresponds to a small carrier self-energy and a small correction of its effective mass, whereas strong coupling is characteristic of materials with

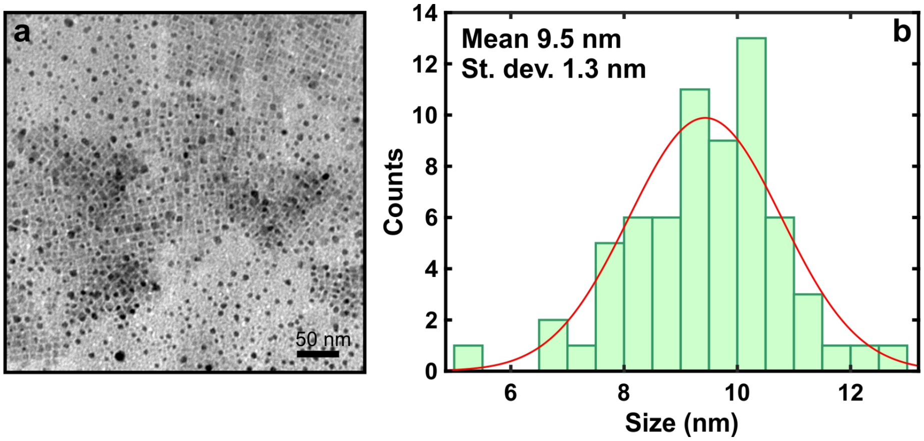

Supplementary Figure 2: TEM image (a) and size analysis (b) of the $CsPbBr_3$ NCs used in the THz-TDS experiment.

a strong dielectric contrast and can result in significant enhancements of the carrier binding energy and effective mass.

The spatial extent of the lattice interaction is given by the polaron radius, r_p, which is defined for each of the two limits of the phonon coupling strength. In the weak coupling limit, the radius is given by the fundamental uncertainty in the carrier position associated with a period of the characteristic lattice vibration:[3, 4]

$$r_p^W = \sqrt{\frac{\hbar}{2m\omega_0}} \tag{1}$$

where m is the bare carrier mass (i.e. in the absence of the polarization cloud) and ω_0 is the lattice frequency. For strong coupling, the polaron radius is found by minimizing the self-energy of the carrier, and can be expressed as:[3]

$$r_p^S = \frac{4\pi^2\hbar^2\bar{\varepsilon}}{me^2} \tag{2}$$

While expressions 1 and 2 are accurate in the limits of weak and strong couping respectively, intermediate values of α (i.e. $\alpha \simeq 1$) require a different approach. Within the Feynman polaron picture, the carrier is approximated as a harmonic oscillator coupled to the crystal lattice. The polaron radius is then the root mean square value of the oscillator position, and the corresponding expression is:[5]

$$r_p = Ar_p^W \tag{3}$$

where A is inversely related to α.

In very polar materials (i.e. strong coupling), there is a significant difference between the short and long-range Coulomb potential, as the dielectric constant changes from ε_S to ε_∞. This induces a self-trapping potential for the carrier,

which represents the long-range carrier-phonon interaction. There is also a short-range interaction, which occurs as the carrier induces a local strain in the lattice in its vicinity. The strain of the lattice modifies the wave function, and hence the energy, of the carrier which produced the deformation. This interaction induces a potential well for the carrier, as nearest ions relax into a new position to lower the energy of the carrier-lattice system. The relative strength of the short and long-range carrier-phonon interactions determines the spatial extent of the polarization induced by the carrier in the lattice and hence the radius of self-trapping. As such, for strong coupling, we distinguish between 'small and 'large' polarons. In the case of a small polaron, the short range interaction dominates, and the length scale of the lattice deformation is comparable to the lattice constant. Small polarons exhibit hopping transport, and are characterized by a low, thermally-activated mobility. In the absence of strong short-range carrier-phonon interaction, the long-range Coulomb interaction polarizes the lattice on a much larger length scale, resulting in a large polaron, which is free to move continuously across the lattice. The mobility then shows an inverse relationship with temperature, as carrier-phonon scattering limits the polaron mean free path.

Measurements of carrier mobility in lead halide perovskites show a temperature dependence $\partial\mu/\partial T < 0$,[6–9] which is indicative of large polarons, and as such we will consider that the short-range carrier-phonon interaction is negligible.

Supplementary Note 3: Selection Rules for First-Order Raman Scattering

In Resonant Raman spectrosopy a sample is illuminated with laser radiation at an energy above the material band gap. When the incoming photons are close to resonance with a real electronic state, strong scattering involving LO phonons can be observed, with intense overtones visible up to very high orders.[10] The overtone progression is produced via the Fröhlich interaction, as excited states couple to the macroscopic electric field of lattice vibrations. The observed spectra can be explained in terms of either a configuration coordinate model, similar to the Franck-Condon principle,[11–13] henceforth referred to as resonant Raman scattering (RRS), or a cascade model, where hot carriers relax via consecutive emissions of single LO phonons, i.e. hot luminescence (HL).[14–16]

The model of multiphonon scattering depends on the material system under consideration. A distinction can be made between free and localized carriers, with regards to the selection rules for first order scattering. Inelastic scattering of light with free excitons in a periodic potential requires strict momentum conservation. At higher orders, wave vector conservation is observed by involving multiple phonons with a total wave vector sum of zero. Conversely, first order scattering (i.e. by one phonon) can only involve phonons of wave vector $\mathbf{k}\cong 0$. In ionic crystals, the dominant scattering mechanism for excited carriers is an intraband Fröhlich scattering, for which the interaction Hamiltonian diverges

at the center of the Brillouin zone.[17] Consequently, first order scattering is essentially forbidden (see also main text). If however the lattice translation symmetry is locally broken, momentum conservation rules are relaxed, and first order inelastic scattering becomes an allowed process. The symmetry breaking may arise from imperfections in the crystal lattice around which carriers can become localized, such as defects or impurities. For a localized carrier the matrix elements for Fröhlich coupling to phonons of wave vector $\mathbf{k} > 0$ are enhanced, as the exciton charge distribution is spread out in reciprocal space.[18] Through the involvement of phonons with non-zero wave vector, first order 'forbidden' LO phonon scattering thus becomes possible. First order scattering involving defects is thereby governed by the same mechanism as the higher order processes, while the free-carrier response remains negligible in comparison.

Supplementary Note 4: RRS versus HL

The models for resonant Raman scattering and hot luminescence are schematically presented in Supplementary Fig. 3, where the vibrational states are represented as the energy levels of simple harmonic oscillators. In RRS, an exciton transitions from a certain vibrational level of its excited state into a different vibrational level of the ground state. The excess energy is released into the lattice as LO phonons. Conversely, in HL, excitons relax into a lower vibrational level of the excited state via consecutive LO phonon emissions followed by relaxation into the ground state via photon emission. In both situations, as long as crystal momentum is conserved, the transition involving a single phonon is forbidden.

In the MPRS experiment discussed in the main text, an indication of the dynamics of phonon emission stems from the FWHM of consecutive phonon lines, Γ, which is inversely related to the scattering lifetime. The implied scattering time in our measurement is $\hbar/\Gamma \sim 10^{-13}$ s (see Figure 1a in main text), which is consistent with typical LO phonon scattering times.[15] It can be therefore assumed that no significant contributions from other broadening mechanisms are present in the observed spectrum. In the case of RRS the FWHM is linked to the anharmonic decay time of the lattice polarization into LO phonons, and is proportional to the number of involved phonons. Consequently, a linear increase in Γ is expected with increasing overtone order.[19] Conversely, in the case of hot luminescence, the width of each consecutive peak is expected to decrease, as successive LO phonon emissions bring carriers closer to the center of the Brillouin zone. The continuously smaller range of accessible wave vectors implies a smaller energy dispersion for the scattered carriers, and a reduced FWHM. The inset to Figure 1a in the main text shows an increasing FWHM with increasing order, and as such the dominant multiphonon emission mechanism is resonant Raman scattering.

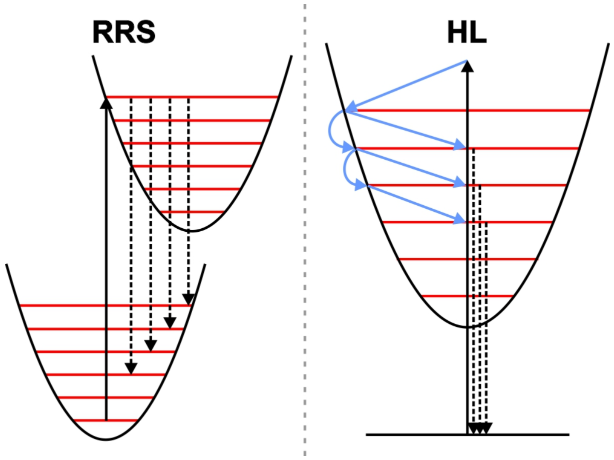

Supplementary Figure 3: Schematic representation of the resonant Raman scattering (RRS) and hot luminescence (HL) processes. The lower and upper parabolas represent the ground and excited excitonic states, respectively. The red lines represent the different vibrational levels of the harmonic oscillators. Solid black arrows represent excitation, blue arrows denote LO phonon emission, and dashed arrows denote PL transitions.

Supplementary Note 5: Comparison between NCs and bulk systems

Within the perovskite family, lead halide perovskite nanocrystals (NCs) are highly regarded for their remarkable light emission properties. They have therefore been intensely studied, with much emphasis placed on their potential optoelectronic applications. On a fundamental level, the physics of perovskite NCs are strongly influenced by the large surface-to-volume ratio of such small particles. This ratio leads to different electronic behavior in NCs compared to bulk materials, since the surface may introduce a significant number of localized states that affect the energy landscape in the material. While a certain degree of surface passivation is achieved by encapsulating perovskite NCs in an organic ligand shell, it has been shown that the attachment of the ligands to the surface is imperfect,[20] resulting in only a partial coverage, that may still allow carriers to become localized. Carrier localization is likely to have an impact on polar exciton-phonon coupling, as the excitonic charge distribution can be modified by the localizing potential, breaking its overall neutrality. Note that a large average electron-hole distance can also reduce the compensation of their respective phonon clouds, thereby producing a non-zero exciton charge distribution. Such a separation may arise due to the presence of an electric field, or if the exciton Bohr radius is simply much larger than the free carrier polaron radii.

Further differences between lead halide perovskites in their bulk and NC form which are relevant to polar exciton-phonon coupling may be stem from

differences in the crystal lattice symmetry between the two systems. A significant change of symmetry can affect the dielectric function and therefore result in a change in the lattice polarity, that will impact the macroscopic electric field of LO phonons. In the case of $CsPbBr_3$ the crystal lattice is orthorhombic from cryogenic temperatures up to well above room temperature[21,22] for both bulk materials and colloidal NCs. As such, the dielectric function is not expected to change significantly, neither between cryogenic and room temperature, nor between the bulk and NC geometries. The NC system at cryogenic temperatures is therefore a good indicator of general exciton-phonon coupling in $CsPbBr_3$, with the caveat that the surface effects which may modify the Fröhlich interaction in NCs are less prominent in bulk single crystals. Polycrystalline thin films, on the other hand, may exhibit behavior more comparable to NCs, due to the increased number of defects at the surface and at inter-grain boundaries, which will have a similar effect as surface-localized states in NCs.

Supplementary Note 6: The Huang-Rhys Factor of Free versus Trapped Carriers, and Non-Adiabatic Effects

In the case of free polarons, the wave vectors available for the carrier-phonon interaction span the range determined by the thermally-available kinetic energy of the heavy carrier, with the maximum value given by:

$$\mathbf{k}_0 = \sqrt{\frac{m^p k_B T}{2\hbar^2}} \tag{4}$$

where m^p is the polaron mass, and $k_B T$ is the thermal energy of the carrier.[18] The Huang-Rhys factor then becomes:

$$S = \sum_{\mathbf{k}<\mathbf{k}_0} \frac{2\pi}{V} \frac{e^2}{\bar{\varepsilon}} \frac{\hbar\omega_0}{\mathbf{k}^2} \left[\hbar\omega_0 + \frac{\hbar^2\mathbf{k}^2}{2m}\right]^{-2} \tag{5}$$

where V is the crystal volume, $\hbar\omega_0$ is the LO phonon energy, and $\bar{\varepsilon}$ is the dielectric contrast, defined in the main text. Note that the Huang-Rhys factor and the Fröhlich constant are intimately linked through the dielectric contrast and the LO phonon energy, thus illustrating how the coupling strength for a particular mode is dependent on the intrinsic strength of the Fröhlich interaction:

$$S = \sum_{\mathbf{k}<\mathbf{k}_0} \frac{2\pi}{V} \alpha \left(\hbar\omega_0\right)^2 \sqrt{\frac{2\hbar}{m\omega_0}} \left[\hbar\omega_0 + \frac{\hbar^2\mathbf{k}^2}{2m}\right]^{-2}, \tag{6}$$

Alternatively, the Huang-Rhys factor can be defined as the square of the total displacement in the phonon coordinate of the excited electronic state (configuration coordinate model).[11]

$$S = \Delta^2 = \sum_{\mathbf{k}} \Delta_{\mathbf{k}}^2. \tag{7}$$

The squared partial displacement corresponding to a certain wave vector $\mathbf{k}$ is:

$$\Delta_{\mathbf{k}}^2 = \frac{2\pi}{V}\frac{e^2}{\hbar\omega_0}\frac{1}{\bar{\varepsilon}}\frac{\rho_{\mathbf{k}}^{*2}}{\mathbf{k}^2}, \tag{8}$$

where $\hbar\omega_0$ is the energy of the phonon mode involved (assumed to be dispersionless), while V is the crystal volume and e is the unit charge. Finally, $\rho_{\mathbf{k}}^*$ represents the Fourier component of the exciton charge distribution, corresponding to the wave vector $\mathbf{k}$.

If either the electron or the hole becomes trapped, we may assume a 1s-like charge distribution, where one carrier is pinned at an imperfection and its counterpart remains bound in its vicinity. The real-space charge distribution is then given by:

$$\rho(\vec{r}) = \frac{1}{\pi a_0^3} e^{-2\frac{|\vec{r}-\vec{r}_0|}{a_0}} - \delta(\vec{r}-\vec{r}_0) \tag{9}$$

At low temperatures, the Fourier components $\rho_{\mathbf{k}}^*$ extend far beyond the thermal limit $\mathbf{k}_0$, and phonon coupling away from the center of the Brillouin zone is extrinsically enhanced, as compared to the intrinsic case of free carriers. Note that while in typical quantum dots the carrier wave functions, and hence the exciton charge distribution, are strongly affected by quantum confinement, this is not true for the current system, because the NCs are significantly larger than the exciton Bohr diameter (11 nm and respectively 4 nm, as estimated from the effective masses and dielectric function of $CsPbBr_3$). Therefore the bulk-like wave-functions considered in this calculation provide a good first-order approximation of the Huang-Rhys factor. With the chosen charge distribution, the S-factor becomes:[11,12]

$$S = \frac{e^2}{a_0\bar{\varepsilon}\hbar\omega_0}\frac{2}{\pi}\int_0^{\frac{\pi}{2}} \frac{x^4(2+x^2)^2}{(1+x^2)^4}dx \tag{10}$$

where a_0 is the exciton Bohr radius, which in our case represents the confinement length scale. The summation over Fourier space is done within the interval $0 \leq \mathbf{k} \leq \mathbf{k}_0 = \frac{\pi}{a_0}$, i.e. the range of available $\mathbf{k}$-vectors for the carrier confined within a distance a_0. Note that in addition to the intrinsic relationship between S and α, Supplementary Equation (10) also contains the extrinsic contribution due to localization, in the form of the charge distribution $\rho(\vec{r})$ which reduces to a proportionality factor upon summation. Interestingly, the Huang-Rhys factor defined in Supplementary Equation (10) above is simply the ratio between an electrostatic potential, proportional to $\frac{e^2}{\bar{\varepsilon}a_0}$, and the energy of the involved LO phonon mode, $\hbar\omega_0$. It thus represents the approximate number of phonons 'contained' in the phonon cloud of the excitonic polaron.

In the absence of carrier localization, low-temperature investigations of photoluminescence and resonant Raman scattering in semiconductor nanocrystals have also demonstrated the influence of non-adiabatic effects on the carrier-phonon interaction.[23,24] Enhanced coupling, beyond the adiabatic free-exciton Huang-Rhys factor, may stem from the Jahn-Teller and pseudo Jahn-Teller effects, when the nanocrystal size is smaller than the exciton dimensions (i.e.

strong quantum confinement). In the experiment described in the main text, the quantum confinement regime of the NCs is weak, meaning that non-adiabatic carrier-phonon coupling is likely negligible and is not considered in our calculations. This approach is sufficient, as demonstrated by the excellent agreement between the experimental and calculated relative cross sections of the M_0 peak and its overtones (Fig. 2b in the main text).

Supplementary Note 7: Modeling the Huang-Rhys Factor

In typical Raman scattering experiments, the energy of the incoming radiation is chosen below the band gap or HOMO-LUMO transition energy of the investigated material. This is done to eliminate the PL background, which is much stronger than the scattered light intensity. When Raman scattering experiments are conducted above the material band gap (RRS), the observed spectrum can differ significantly from the non-resonant case. In an RRS experiment, the energy of the incoming radiation corresponds to that of excited electronic states (i.e. excitons) in the material. Each electronic state can also be vibrationally excited. The vibrational excitation represents the number of phonons which are present in the electronically-excited state, and which are emitted in the scattering event. The resonance condition for the RRS experiment is given by the denominator of eq. (6) in the main text. The cross-section for allowed scattering increases when the energy of incoming light is resonant with a vibrational level of an electronically excited state. In the case of NCs, the energy of electronic states varies between particles, and therefore a certain distribution is assumed. This means that only a subset of the NCs will be in resonance with the incoming photons, and in order to correclty estimate the scattering cross-section for the experiment, contributions corresponding to all exciton energies must be added together.

In modeling the Huang-Rhys factor from the MPRS data, the total Raman cross section is:[12]

$$\sigma_{tot}^{(n)} = \int \sigma_E^{(n)} G(E_g, E_g^0, \sigma) dE, \tag{11}$$

where $G(E_g, E_g^0, \sigma)$ is the normal probability distribution function centered at E_g^0 with variance σ. The real distribution of E_g is similar to the PL lineshape of the NC ensemble shown in Figure 1c of the main text. However, inter-NC energy transfer, charging, and other such effects alter the energy distribution of emitted photons with respect to the real density of NC states. The Huang-Rhys factor corresponding to the multiphonon progression also depends on the damping factor Γ_0, which represents the linewidth of a single NC. This parameter has been determined experimentally.[25,26] Within a first-order approximation, a choice of $\Gamma_0 = 1$ meV is sufficiently accurate. For this value, and using $E_g^0 = 2.375$ eV and $\sigma = 32.5$ meV, we obtain $S \simeq 0.39$, and the relative intensities of the multiphonon lines closely match the experimental data, as shown in Figure 2b in the main text.

The Huang-Rhys factor agrees with the previous experimental value,[27] whereas the value of σ is somewhat larger than the variance of the low-temperature inhomogeneous PL of the NC sample, but remains comparable to it, as expected. Similarly, the center of the distribution of transition energies is realistically close to the position of the NC PL band.

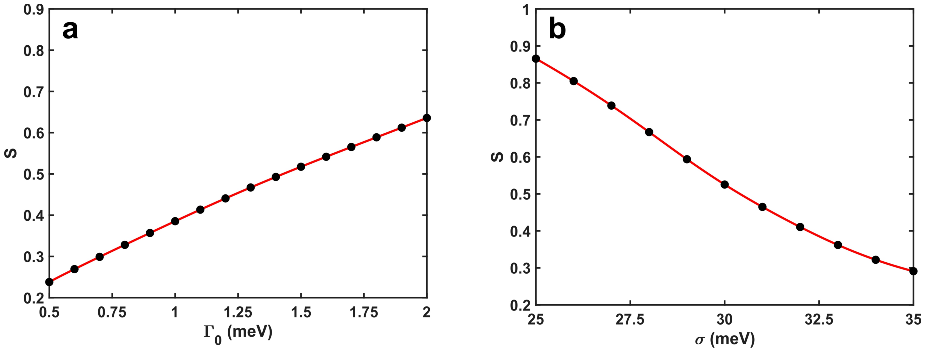

Supplementary Figure 4: Dependence of the Huang-Rhys factor a) on the spread in excitonic transition energies, given by the variance σ of a Gaussian distribution (when $\Gamma_0 = 1$ meV), and b) on the homogeneous broadening represented by the damping factor Γ_0 (for $\sigma = 32.5$ meV) (b). The value of $E_g^0 = 2.375$ eV was used in both (a) and (b). The red lines are guides to the eye.

Supplementary Fig. 4 shows the change in S as functions of σ and Γ_0, respectively. From the figure it is evident that, as the parameters are varied within the spread of experimental values, the Huang-Rhys factor does not change significantly, and the obtained value remains accurate within a first order approximation.

Supplementary Note 8: Terahertz Time-Domain Spectroscopy

Measurements were conducted on a high density colloidal suspension of $CsPbBr_3$ nanocrystals in hexane. The NCs were synthesized via the method described by De Roo et al.[20] TEM analysis reveals a size of roughly 9.5 nm. The sample was placed in a quartz cuvette, with an optical path length $d = 4$ mm, as shown in Supplementary Fig. 5.

The THz-TDS measurement consists of sending a focused THz pulse through the sample and comparing the incoming and transmitted signal. The time-resolved signal of the transmitted THz pulse is shown in Supplementary Fig. 6. The signal transmitted through air is considered the incoming pulse, and we obtain the refractive index and extinction coefficient as functions of the phase shift and relative absorption of the pulse transmitted through the sample of interest. By also measuring the contribution of hexane alone, we can extract the

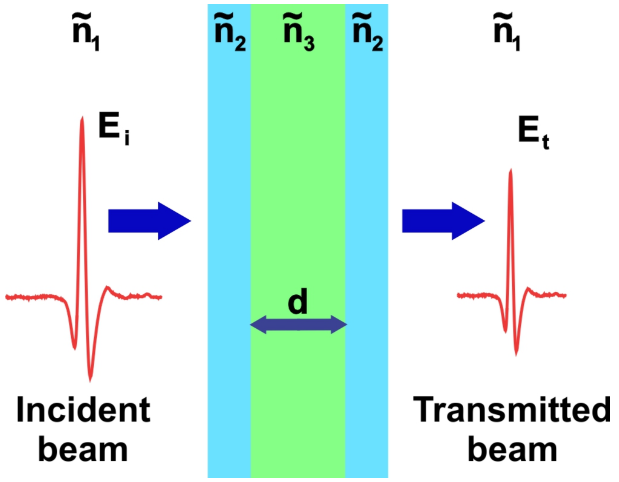

Supplementary Figure 5: THz-TDS measurement of a cuvette containing a suspension of $CsPbBr_3$ NCs. The amplitude and phase of the transmitted beam depends on the interfaces and distance travelled through the media $\tilde{n}_2$ and $\tilde{n}_3$.

refractive index, n, and the extinction coefficient, κ, of the $CsPbBr_3$ NCs.

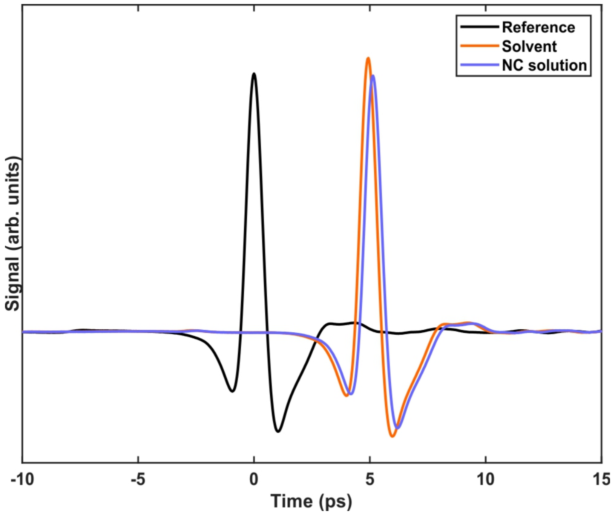

Supplementary Figure 6: Time-domain THz signal, corresponding to the empty sample cuvette (Air), hexane reference sample (Solvent), and the sample of interest (NC solution).

We measured the transmitted THz pulse through a suspension of $CsPbBr_3$

NCs in hexane. The transfer function of the sample is defined as:

$$\hat{T}(\omega) = \frac{\hat{E}_t^{sample}(\omega)}{\hat{E}_t^{ref}(\omega)} \tag{12}$$

where $\hat{T}(\omega)$ and $\hat{E}_t(\omega)$ are Fourier transforms of the time-dependent transfer function and time-resolved THz signal that we measure. In order to calculate $\hat{T}(\omega)$, a reference measurement is conducted, of the same quartz cuvette containing a known substance (in this case, an empty cuvette). The analytic definition of $\hat{T}(\omega)$ is:

$$\hat{T}(\omega) = \frac{\hat{E}_t(\omega)}{\hat{E}_t^{ref}(\omega)} = \frac{\hat{t}_{23}\hat{t}_{32}}{\hat{t}_{23}^{ref}\hat{t}_{32}^{ref}} e^{i\left(\tilde{n}_3 - \tilde{n}_3^{ref}\right)\frac{\omega d}{c}} \tag{13}$$

where $\hat{t_{ij}}$ are Fresnel transmission coefficients, with the subscript indices corresponding to the media defined in Supplementary Fig. 5. By solving for the absolute value and argument of the complex valued function, the complex refractive index $\tilde{n} = n + i\kappa$ of the NC suspension is obtained.

In order to consider only reliable data, the analysis is restricted to regions where the transmitted signal-to-noise ratio (SNR) is sufficiently high. As such, a trusted range of frequencies can be defined. Supplementary Fig. 7 shows the Fourier transformed transmitted signals corresponding to the three different samples. The shaded areas correspond to frequencies outside the trusted range, where the calculated SNR is less than 25 dB. The trusted frequency range is then between 0.2 and 1.7 THz.

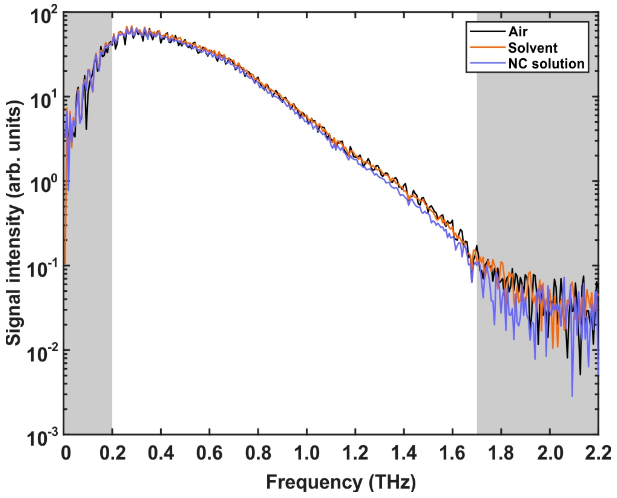

Supplementary Figure 7: Frequency-domain amplitude of the THz signal, corresponding to the empty cuvette (Air), hexane reference sample (Solvent), and the sample of interest (NC solution). The shaded areas mark frequencies outside of the trusted range.

To determine the dielectric function of $CsPbBr_3$, effective medium theory is used to separate the contributions of the solvent and the NCs. The dielectric function of the solvent is determined in the same way as that of the NC solution. The dielectric function of $CsPbBr_3$ is determined by considering the NCs as polarizable spheres of volume V, having a polarizability given by:[28]

$$\alpha_{NC} = 3\varepsilon_{solv} V \left(\frac{\varepsilon_{NC} - \varepsilon_{solv}}{\varepsilon_{NC} + 2\varepsilon_{solv}} \right) \tag{14}$$

where ε_{NC} is the dielectric function of the particle and ε_{solv} corresponds to the medium in which it is dispersed. We introduce this polarizability into the Clausius-Mossotti relation, which expresses the dielectric function of a material in terms of the polarizability of its constituent atoms or molecules:

$$\frac{\varepsilon_{NC} - 1}{\varepsilon_{NC} + 2} = \frac{N\alpha_{NC}}{3} \tag{15}$$

obtaining the equation for the effective dielectric function of a composite material containing polarizable inclusions, known as the Maxwell-Garnett equation:

$$\frac{\varepsilon_{eff} - \varepsilon_{solv}}{\varepsilon_{eff} + 2\varepsilon_{solv}} = f \frac{\varepsilon_{NC} - \varepsilon_{solv}}{\varepsilon_{NC} + 2\varepsilon_{solv}} \tag{16}$$

Here, f corresponds to the volume fraction of the solution occupied by the NCs, and ε_{eff} is the effective dielectric function which is obtained from measurements. In this approximation, the Maxwell-Garnett equation estimates the dielectric response of a suspension of polarizable spheres. Although at large contrasts between the NC and solvent dielectric functions the spherical assumption fails, the range of reported dielectric function values for $CsPbBr_3$[1,29] suggests that the assumption is quite accurate, with an underestimation of the particle polarizability of only $\sim 5\%$.[28] The volume fraction 1.8 ± 0.27 % is obtained by weighing the residue of a known volume of NC solution after evaporation of the solvent. Note that the reported volume fraction corresponds strictly to the $CsPbBr_3$ inclusions, without the contribution of the organic ligand shell, which has a similar response as the organic solvent. By solving the Maxwell-Garnett equation we find the real and imaginary parts of the frequency-dependent dielectric function of $CsPbBr_3$, as shown in the main text.

The relative error in the calculated dielectric function can be obtained by propagating the noise in the THz signal, and the result is shown in Supplementary Fig. 8. The relative error within the trusted range of frequencies is below 1%.

The results obtained here represent the room-temperature dielectric behavior of $CsPbBr_3$. In the main text, the Fröhlich constant is calculated using the dielectric contrast, and correlated to the low-temperature phonon coupling behavior. It has been shown that $CsPbBr_3$ crystallizes in the orthorhombic *Pnma* space group both at room temperature[30,31] and at cryogenic temperatures.[27] The absence of crystal phase transitions between cryogenic and room temperature suggests that the dielectric function does not change significantly in between, and the use of the room temperature dielectric function in the interpretation of low temperature phonon coupling is therefore valid.

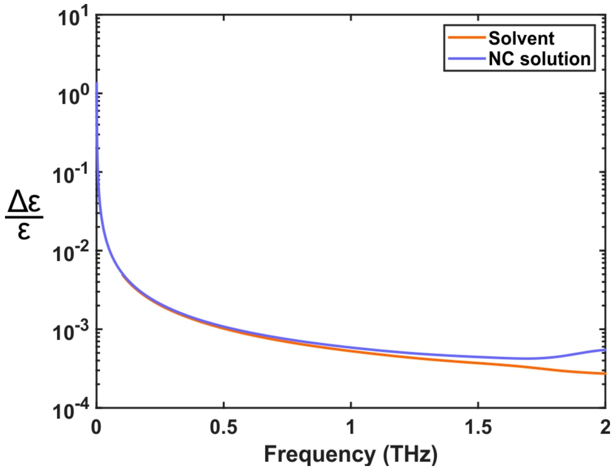

Supplementary Figure 8: Relative error in the calculated dielectric function.

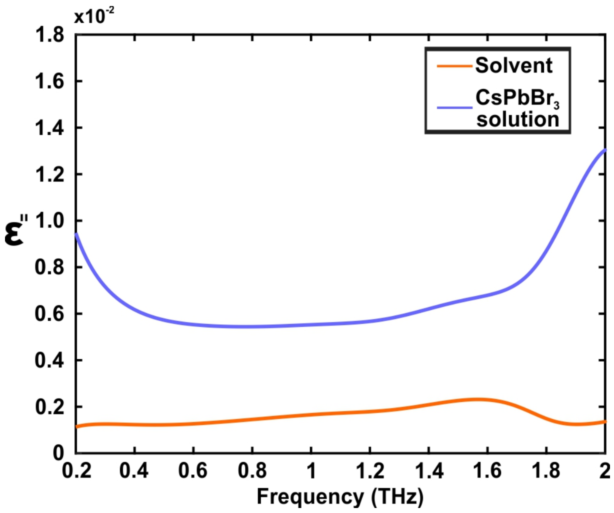

Supplementary Figure 9: Imaginary part of the dielectric response of the $CsPbBr_3$ NCs compared to that of the solvent. The low-frequency relaxation and the Lorentz resonance are only observed in the presence of $CsPbBr_3$ NCs.

Supplementary References

[1] L. Protesescu, S. Yakunin, M. I. Bodnarchuk, F. Krieg, R. Caputo, C. H. Hendon, R. X. Yang, A. Walsh, and M.V. Kovalenko. Nanocrystals of cesium lead halide perovskites (cspbx$_3$, x = cl, br, and i): Novel optoelectronic

materials showing bright emission with wide color gamut. *Nano Lett.*, 15:3692–3696, 2015.

[2] M. Bruzzi, N. Falsini, N. Calisi, and A. Vinattieri. Electrically active defects in polycrystalline and single crystal meta halide perovskite. *Energies*, 13:1643, 2020.

[3] J. T. Devreese. Polarons. *Encyclopedia of Applied Physics*, Vol.14:pp. 383–409, 1996. Wiley-VCH Publishers, Inc.

[4] D. Emin. *Polarons.* Cambridge University Press, 2013. isbn: 978-0-521-51906-9.

[5] T. D. Schultz. Slow electrons in polar crystals: Self-energy, mass, and mobility. *Phys. Rev.*, 116:526–543, 1959.

[6] X. Y. Zhu and V Podzorov. Charge carriers in hybrid organic-inorganic lead halide perovskites might be protected as large polarons. *J. Phys. Chem. Lett.*, 6(23):4758–4761, 2015.

[7] R. L. Milot, G. E. Eperon, H. J. Snaith, M. B. Johnston, and L. M. Herz. Temperature-dependent charge-carrier dynamics in $ch_3nh_3pbi_3$ perovskite thin films. *Adv. Funct. Mater.*, 25:6218–6227, 2015.

[8] M. Karakus, S. A. Jensen, F. D'Angelo, D. Turchinovich, M. Bonn, and E. Cánovas. Phonon-electron scattering limits free charge mobility in methylammonium lead iodide perovskites. *J. Phys. Chem. Lett.*, 6:4991–4996, 2015.

[9] K. Miyata, D. Meggiolaro, M. T. Trinh, P. P. Joshi, E. Mosconi, S.C. Jones, F. De Angelis, and X.-Y. Zhu. Large polarons in lead halide perovskites. *Sci. Adv.*, 3(8):e1701217, 2017.

[10] R. C. C. Leite, J. F. Scott, and T. C. Damen. Multiple-phonon resonant raman scattering in cds. *Phys. Rev. Lett.*, 22(15):780, 1969.

[11] R. Merlin, G. Güntherodt, R. Humphreys, M. Cardona, R. Suryanarayanan, and F. Holtzberg. Multiphonon processes in ybs. *Phys. Rev. B*, 17(12):4951, 1978.

[12] M. C. Klein, F. Hache, D. Ricard, and C. Flytzanis. Size dependence of electron-phonon coupling in semiconductor nanospheres: The case of cdse. *Phys. Rev. B*, 42(17):11123, 1990.

[13] A. P. Alivisatos, T.D. Harris, P.J. Carroll, M. L. Steigerwald, and L. E. Brus. Electron-vibration coupling in semiconductor clusters studied by resonance raman spectroscopy. *J. Chem. Phys.*, 90(7):3463, 1989.

[14] A. Garcìa-Cristòbal, A. Cantarero, C. Trallero-Giner, and M. Cardona. Excitonic model for second-order resonant raman scattering. *Phys. Rev. B*, 49(19):13430, 1994.

[15] R. M. Martin and C. M. Varma. Cascade theory of inelastic scattering of light. *Phys. Rev. Lett.*, 26(20):1241, 1971.

[16] R. Zeyher. Theory of multiphonon raman spectra above the energy gap in semiconductors. *Solid State Commun.*, 16:49–52, 1975.

[17] P. Y. Yu and M. Cardona. *Fundamentals of Semiconductors.* Springer Verlag-Berlin Heidelberg, 1999. isbn: 978-3-662-03850-5.

[18] J. J. Hopfield. A theory of edge-emission phenomena in cds, zns and zno. *J. Phys. Chem. Solids*, 10:110–119, 1959.

[19] T. P. Martin. Multiple-order raman scattering by a localized mode. *Phys. Rev. B*, 13(8):3617, 1976.

[20] J. De Roo, M. Ibàñez, P. Geiregat, G. Nedelcu, W. Walravens, J. Maes, J. C. Martins, I. Van Driessche, M. V. Kovalenko, and Z. Hens. Highly dynamic ligand binding and light absorption coefficient of cesium lead bromide perovskite nanocrystals. *ACS Nano*, 10:2071–2081, 2016.

[21] I. Mannino, G.and Deretzis, E. Smecca, A. La Magna, A. Alberti, D. Ceratti, and D. Cahen. Temperature-dependent optical band gap in $cspbbr_3$ $mapbbr_3$, and $fapbbr_3$ single crystals. *J. Phys. Chem. Lett.*, 11:2490–2496, 2020.

[22] B. T. Diroll, H. Zhou, and R. D. Schaller. Low-temperature absorption, photoluminescence, and lifetime of $cspbx_3$ (x = cl, br, i) nanocrystals. *Adv. Funct. Mater.*, 28:1800945, 2018.

[23] V. M. Fomin, V. N. Gladilin, J. T. Devreese, E. P. Pokatilov, S. N. Balaban, and S. N. Klimin. Photoluminescence of spherical quantum dots. *Phys. Rev. B*, 57:2415–2425, 1998.

[24] E. P. Pokatilov, S. N. Klimin, V. M. Fomin, J. T. Devreese, and F. W. Wise. Multiphonon raman scattering in semiconductor nanocrystals: Importance of nonadiabatic transitions. *Phys. Rev. B*, 65:075316, 2002.

[25] G. Rainò, G. Nedelcu, L. Protesescu, M. Bodnarchuk, M. V. Kovalenko, R. F. Mahrt, and T. Stöferle. Single cesium lead halide perovskite nanocrystals at low temperature: Fast single-photon emission, reduced blinking, and exciton fine structure. *ACS Nano*, 12:2485–2490, 2016.

[26] M. A. Becker, R. Vaxenburg, G. Nedelcu, P. C. Sercel, A. Shabaev, M. J. Mehl, J. G. Michopoulos, S. G. Lambrakos, N. Bernstein, J. L. Lyons, T. Stöferle, R. F. Mahrt, M. V. Kovalenko, D. J. Norris, G. Rainò, and A. L. Efros. Bright triplet excitons in caesium lead halide perovskites. *Nature*, 553:189–193, 2018.

[27] C. M. Iaru, J. J. Geuchies, P. M. Koenraad, D. Vanmaekelbergh, and A. Yu. Silov. Strong carrier-phonon coupling in lead halide perovskite nanocrystals. *ACS Nano*, 11(11):11024–11030, 2017.

[28] A. Sihvola, P. Ylä-Oijala, S. Järvenpää, and J. Avelin. Polarizabilities of platonic solids. *IEEE Trans. Antennas Propag.*, 52:2226–2233, 2004.

[29] G. R. Yettapu, D. Talukdar, S. Sarkar, A. Swarnkar, A. Nag, P. Ghosh, and P. Mandal. Terahertz conductivity within colloidal cspbbr$_3$ perovskite nanocrystals: Remarkably high carrier mobilities and large diffusion lengths. *Nano Lett.*, 16:4838–4848, 2016.

[30] M. S. Kirschner, B. T. Diroll, P. Guo, S. M. Harvey, W. Helweh, N. C. Flander, A. Brumber, N. E. Watkins, A. A. Leonard, A. M. Evans, M. R. Wasielewski, W. R. Dichtel, X. Zhang, L. X. Chen, and R. D. Schaller. Photoinduced, reversible phase transitions in all-inorganic perovskite nanocrystals. *Nat. Commun.*, page 10:504, 2019.

[31] P. Cottingham and L. Brutchey. On the crystal structure of colloidally prepared cspbbr$_3$ quantum dots. *Chem. Commun.*, 52:5246, 2016.
